# Deleveraging and decapacity: A comparative analysis of corporate capital allocation based on asset reversibility

**DOI:** 10.1371/journal.pone.0291350

**Published:** 2023-11-15

**Authors:** Songbo Jia, Chenying Sang, Shiwei Su, Jianxiang Xu

**Affiliations:** 1 School of Finance, Henan University of Economics and Law, Zhengzhou, China; 2 School of Finance, Zhongnan University of Economics and Law, Wuhan, China; The University of Sydney, AUSTRALIA

## Abstract

China’s stimulus policies have caused overleveraging and overcapacity for the sustainable development of most industries (particularly high-pollution and energy-intensive industries). Thus, deleveraging and decapacity have become the two best options for the above industries to achieve long-term sustainable development. Based on China’s A-share listed companies from 2009 to 2019, this study investigated the effect of deleveraging and decapacity on corporate capital allocation using fixed effects, propensity score matching (PSM) and difference-in-differences (DID). A homogeneity analysis of geographical and firm characteristics was also conducted. The results show that: (1) Deleveraging and decapacity can significantly increase financial capital allocation by 3.67%, and decapacity can increase investment-related capital allocation by 0.63%. This indicates asset allocation optimization for sustainable development. (2) High asset reversibility can weaken the effect of deleveraging on financial capital allocation while strengthening the effect of decapacity on capital investment. (3) The impact of deleveraging and decapacity may vary among companies due to heterogeneous asset reversibility resulting from geographical locations and technological intensities. Given the current global energy crisis, optimizing capital allocation has become essential in addressing resource shortages and achieving long-term sustainable development. This study may provide a reference for alleviating corporate capital misallocation.

## 1. Introduction

Since the global financial crisis erupted in 2008, China’s central and local governments implemented economic stimulus policies to mitigate the impact of external shocks on the economy. These policies increased the leverage ratio of the real economy and stimulated the growth of industries with overcapacity, thereby posing significant challenges. As the global economy stabilizes, the Chinese government continues to address overcapacity and overleveraging resulting from the financial crisis, particularly in high-pollution and energy-intensive industries. Since the launch of the "supply-side structural reform" from 2015, China’s latest outline of the 14th Five-Year Plan in 2021 also emphasized the government’s commitment to enhance long-term mechanisms for resolving overcapacity and maintaining a stable leverage ratio. This reaffirms the significance of deleveraging and decapacity as key objectives of China.

**Fig 1:**
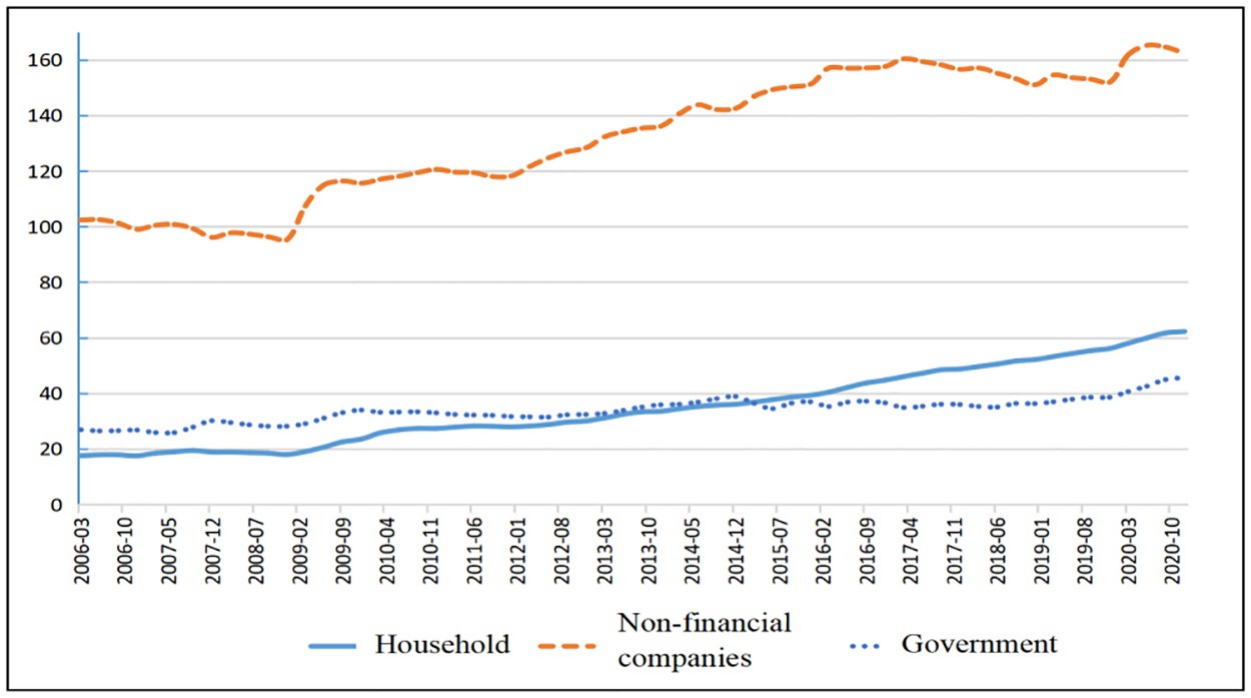


How can we reduce overcapacity? In addressing the structural optimization challenges faced by non-financial enterprises, it is essential to recognize that deleveraging alone has limited effects. The root cause lies in the need to optimize structural capacity on the supply side of the economy. Therefore, the essence of deleveraging lies in reducing excess capacity. Fig 1 illustrates that high leverage mainly occurs in China’s non-financial corporate sectors.

Fig 1 illustrates that the non-financial corporate sector in China has a significantly higher leverage ratio than other sectors. This suggests that deleveraging policies implemented as part of China’s supply-side reform have been focused on structural deleveraging within the non-financial corporate sector. Overleveraging and overcapacity reflect the irrational development of the real economy, hindering the optimization of corporate and social benefits through effective capital allocation. Therefore, studying the effects of deleveraging and decapacity on the economy has both theoretical and practical significance for improving corporate capital allocation.

It has been demonstrated that reducing overcapacity can significantly alleviate distortions in production factors, enhance total factor productivity (TFP) and increase return on investments (Zhang and Zhang [1]; Song *et al*. [2]). This, in turn, can facilitate industrial upgrading and technological advancement. Even before the 12th Five-Year Plan (2011–2015), China had initiated efforts to reduce production capacity in industries with severe overcapacity, such as steel and cement. However, with the policies being implemented, it becomes crucial to explore the extent to which deleveraging and decapacity can effectively impact corporate capital allocation efficiency and the differences in their impacts. In addition, it is important to understand transmission mechanisms underlying these effects. These questions, among others, require further discussion.

This study focused on China’s A-share listed companies as the research subject and employed various empirical methods, such as the fixed-effect model, propensity score matching (PSM) and difference-in-differences (DID) analysis, to examine the effects of deleveraging and overcapacity on corporate investment. In addition, how asset reversibility can influence the impact of corporate capital investment was investigated. Furthermore, homogeneity analysis was also conducted on regional and corporate characteristics. The findings reveal that as capacity reduction increased, enterprises tended to hold more financial assets. Moreover, the level of asset reversibility can affect the impact of deleveraging on corporate financial capital allocation. Higher reversibility can reduce the impact of deleveraging while strengthening the impact of decapacity on corporate capital investment. Existing studies primarily focus on the impact of factors on corporate capital allocation, such as economic policy uncertainty (Huang *et al*. [3]; Gao *et al*. [4]), corporate social responsibility (Bhandari and Javakhadze [5]), political ideology (Kempf *et al*. [6]; Hu and Xu [7]), environmental regulation (Liu and Liu, 2022 [8]) and industrial policy (Zhou and Zhao [9]). However, corporate capital allocation has rarely been studied from the perspective of actual corporate operations. Deleveraging can enhance corporate financial flexibility and address issues of over-investment and under-investment (Ling and Wu [10]). Decapacity can release excess capital, equipment and labor resources, improving corporate innovation activities and investment (Ma *et al*. [11]). These can contribute to asset appreciation (Li *et al*. [12]). Therefore, it is crucial to examine whether and how deleveraging and decapacity can impact financial and investment-related asset allocation within companies. Such analysis is valuable for achieving a rational capital allocation, improving the capital allocation efficiency and thus economic growth.

This paper can contribute to the existing literature in three ways: (1) It examines the determinants of corporate capital allocation by considering corporate leverage and capacity utilization, highlighting the significance of capital allocation for long-term sustainable economic development. Many developing countries are challenged by the middle-income trap. Thus, understanding the driving forces to overcome this trap is crucial for their sustainable development. Previous studies have primarily focused on corporate investment efficiency and have explored how capital investment affects economic development. However, studies based on the endogenous growth theory emphasize that a country’s long-term economic growth depends not only on the high savings rate resulting from the constant marginal product of capital, but also on effective capital allocation (Bovenberg and Smulders [13]; Kraay and McKenzie [14]). (2) Overleveraging and overcapacity encountered by China may also occur in other developing countries during their transition to developed economies (Eichengreen *et al*. [15]; Bulman *et al*. [16]). China has extensive experience in addressing economic transition, and its deleveraging and decapacity policies have practical significance for other developing countries (particularly those in Eastern Europe, Latin America and Southeast Asia). (3) This study focuses on the micro-level effects of deleveraging and decapacity on asset allocation and introduces the concept of asset reversibility to enhance the framework of corporate asset allocation. Asset reversibility, to some extent, can reflect a corporation’s risk resilience (Bernanke, 1983 [17]; Pindyck, 1991 [18]; Gulen and Ion, 2016 [19]). Therefore, our study can shed light on the strategic choices made by corporations regarding financial and investment-related asset allocation when facing risks. This can provide valuable insights during the current global economic recession.

## 2. Literature and research hypothesis

### 2.1 Literature review

In terms of the effects of deleveraging and decapacity on corporate investment and the impact of asset reversibility on corporate capital allocation, existing literature can be categorized into three main groups.

#### i. Relationships between cooperate leverage and corporate investment

Rapid credit growth rates and significantly increasing financial leverage are commonly leading indicators of financial crises and pose risks to long-term economic growth (Schularick and Taylor [20]). The impact of leverage on corporate investment decisions has been extensive discussed in the field of corporate finance (Kenc and Driver [21]).

An excessive leverage ratio can increase an enterprise’s risk of credit default and bankruptcy. Enterprisees with high leverage ratios commonly experience weak profitability, low interest coverage and poor liquidity. Due to external economic challenges, cash flow problems can rapidly escalate into balance sheet issues. Specifically, higher interest expenses associated with high leverage ratios can divert available funds away from fixed asset investment, undermining social capital formation and economic growth (Liu *et al*. [22]). Alexandridis *et al*. [23] suggested that excessive debt can impede a company’s ability to raise funds, thereby affecting its investment decisions. Shahzad *et al*. [24] examined the impact of loan growth on financial health using the two-step generalized method of moments (GMM) estimation technique. They found that non-performing loans directly impaired balance sheet expansion, reduced liquidity and had a significant positive relationship with rapid leverage growth. The rise in non-performing loans is a critical feature of financial crises and threatens the stability and sustainability of the financial system.

Furthermore, excessive leverage can lead companies to underinvest. Myers’ debt overhang theory suggests that higher leverage can increase the likelihood that a company abandons positive net present value projects in the future [25]. Based on the panel data from listed companies between 2006 and 2015, Vo [26] conducted panel data analysis using fixed-effect econometric techniques and found a negative correlation between leverage and investment. This implies that debt can constrain corporate investment, and highly leveraged companies tend to reduce future investments, thereby diminishing corporate value.

Deleveraging can encourage enterprises to invest in capital assets and achieve sustainable operations. Overleveraging problems in Chinese companies are primarily caused by excessive capacity and flawed financing structures (Ma and Laurenceson [27]). Selecting capital structure can be considered one of the most critical decisions faced by managers. Changes in leverage ratios can impact a company’s financing ability, risk profile, cost of capital, investment decisions, strategic choices and ultimately shareholder wealth (Cai and Zhang [28]). Moreover, companies exhibit significant differences in their sensitivity to capital and market value of cash holdings during leverage adjustment (Denis and Sibilkov [29]; Faulkender and Wang [30]). Companies, driven by capital arbitrage, tend to allocate some financial assets to alleviate financing constraints and to mitigate the volatility of industrial investments due to their high liquidity and profitability (Demir [31]). In addition, the nature of companies can also affect deleveraging. Deleveraging state-owned enterprises (SOEs) can enhance the efficiency of local funds (Zhang *et al*. [32]), while non-SOEs tend to stimulate investment through deleveraging. High leverage in non-financial companies has severely impeded corporate investment, and deleveraging can encourage investment in capital assets (Liu [33]).

Furthermore, Zhu *et al*. [34] showed that enterprises can promote innovation and strive for sustainable operations by mitigating financialization-associated capital crowding effects and reducing agency costs. Deleveraging policies can restrain the over-investment of debt-burdened SOEs and alleviate the under-investment of debt-laden non-SOEs (Ling and Wu [10]). Some researchers support the role of deleveraging in terms of investment efficiency. For instance, Lou [35] found that deleveraging by non-financial enterprises should consider structural characteristics, as leverage ratios vary significantly across regions, industries and departments. Targeted structural deleveraging can optimize investment structures and enhance capital utilization efficiency. Lai *et al*. [36] also demonstrated that the implementation of China’s deleveraging policies reduced the leverage ratio of SOEs while improving their investment efficiency and resource allocation.

#### ii. Relationships between decapacity and corporate investment

Since 2013, overcapacity in China’s coal, cement, steel and other industries has become increasingly severe, significantly and negatively impacting resource allocation and the national economy. Most of the existing literature has examined the effects of overcapacity reduction on enterprise investment from the perspectives of TFP, technological change and asset structure.

Decapacity initiatives can enhance enterprises’ TFP and profitability. Zhang *et al*. [37] analyzed the survey data of Chinese enterprises provided by the World Bank and found that increasing capacity utilization rates can significantly and positively affect enterprises’ TFP. Zhang *et al*. [38] utilized the Super-SBM-Malmquist index method to measure TFP, technological change and efficiency change in China’s coal industry. Based on a regression discontinuity (RD) design, they revealed that the decapacity policy implemented after 2016 significantly stimulated TFP growth and technological change in coal enterprises. The fundamental difference between "Made in China" and "Created in China" lies in whether companies choose to invest in capacity expansion or technological innovation during investment (Sun and Dong [39]). Using DID, Tian *et al*. [40] found that decapacity policies can substantially enhance the profitability of Chinese industries burdened with overcapacity, such as steel and coal. This can be achieved by reducing companies’ period costs (i.e., management, operating and selling expenses) while improving labor returns and gross profit margins.

Decapacity initiatives can restructure and optimize enterprises’ asset business structures, thereby improving business performance and accelerating asset turnover. Zhu and Zhu [41] collected the data of overcapacity reduction indicators in the coal industry and asset structure changes of 34 listed coal companies from 2009 to 2018. Their findings demonstrated that, under the environmental pressure of overcapacity reduction, listed coal companies tended to eliminate outdated production capacity, improve coal processing and conversion rates and conduct rational capital investment allocations. These operations can accelerate the turnover of fixed and current assets and facilitate the orderly arrangement of the asset structure, the non-coal business expansion and the development of clean, low-carbon, efficient, and high-quality energy resources. Reducing overcapacity can help to adjust the proportion of the virtual and real economy, optimize investment structure and enhance investment efficiency (Wang and Lu [42]). This is consistent with the conclusions of Yang *et al*. [43].

Decapacity efforts can also facilitate resource allocation adjustment in enterprises and contribute to technological innovation. Overcapacity can result in a large portion of production factors, such as labor, land, capital, plants and equipment. These factors are occupied by "zombie enterprises" that are unable to effectively participate in emerging industries, inducing substantial resource misallocation and waste. Supply-side adjustments exhibit stickiness and hysteresis, making it difficult for production factors to transfer from areas of ineffective demand to areas of effective demand and from low-end fields to middle and high-end fields. The phase of "rashly putting up establishments" in the manufacturing industry’s development has generally ended, and industrial development now mainly relies on upgrading the industrial value chain and increasing the product-added value (Wang [44]). Excessive capacity of enterprises occupies scarce resources (such as capital, equipment, and labor force) that could have been utilized for innovation activities, resulting in a crowding-out effect on innovation investment (Zhao *et al*. [45]). Decapacity initiatives can release these occupied resources and mitigate the crowding-out effect on innovation activities. These resources then serve as a foundation for innovation activities, thereby increasing innovation investment (Ma *et al*. [11]). Overall, reducing overcapacity can increase enterprise profits, optimize capital flows between departments, promote asset appreciation within the enterprise and other departments and thus reduce the enterprise’s asset-liability ratio and debt risk (Liu [33]; Li *et al*. [12]).

#### iii. Relationships between asset reversibility and corporate asset allocation

In terms of asset reversibility measurement, scholars initially used the proportion of fixed assets as an index (Balakrishnan and Fox [46]; Gulen and Ion [19]). Subsequently, Kim and Kung [47], Beutler and Grobety [48] and Liu and Zhang [49] expanded the understanding of asset reversibility. Considering different types of businesses, industry attributes and asset categories, Kim and Kung [47] calculated asset reversibility utilizing input-output tables of the national economy and the data from listed companies. They found that variations in asset reversibility among different companies can lead to diverse asset allocations. This approach provides a more specific definition of asset reversibility and encompasses the saleability of more than 180 different asset classes. Compared with other measures that treat all assets uniformly, this approach can capture cross-industry saleability, which becomes crucial when peer companies within an industry experience low demand during industry shocks.

The impact of asset reversibility on enterprise asset allocation can be clarified from the perspective of realized value and production factors. From a realized value perspective, when the level of asset convertibility is low, enterprise assets tend to have lower liquidation transaction prices and higher disposal costs. This can result in a loss of realized value and significant sunk costs for internal and external investments (Cantillo and Wright [50]; Chen *et al*. [51]). Rong *et al*. [52] argued that asset redeployability was vital for investment and equity value in companies facing financing constraints. Therefore, managers should consider asset characteristics and liquidation value when engaging in asset transactions. Liu *et al*. [53] noted that asset reversibility can affect the relationship between economic policy uncertainty and fixed asset investment. In an environment with increased economic policy uncertainty, project success probabilities decrease, while default risks and the cost of debt financing increase. Companies tend to hold more cash to manage financial distress caused by cash flow uncertainty.

In terms of production factors, low asset reversibility implies that enterprises possess non-convertible assets. Thus, it is challenging to timely reallocate production factors from non-tradable to tradable activities, especially when high non-repayable debts cannot be effectively resolved (Loublier [54]). This rigidity in capital allocation can increase the costs associated with internal capital allocation, hinder efforts to overcome industry barriers and prevent the transfer of occupied resources to other business activities. Furthermore, Qi *et al*. [55] found that asset reversibility was crucial to understand the impact of economic policy uncertainty on corporate investment. Specifically, with greater corporate asset irreversibility, the investment processing cost, the opportunity cost of current cross-border mergers and acquisitions and the risk associated with potential future investments increase.

It is evident that excessive debt limits a company’s investment in its core business activities. Appropriate deleveraging can help restore financial adequacy, optimize investment structures and improve capital efficiency. Overcapacity can lead to significant resource misallocation, waste and risk aggregation. However, overcapacity reduction policies can enhance the TFP of enterprises, adjust the balance between the virtual and real economy, promote technological innovation, and restructure and optimize business asset structures. Asset reversibility, as a critical corporate characteristic, can significantly influence the investment and financing decisions of companies.

To better summarize the main findings of the existing literature, Table 1 presents the studies on the impact of deleveraging and decapacity relevant to this paper.

**Table 1:** Summary of the impact of deleveraging and decapacity.

Example	Topic	Theoretical basis	Key findings or propositions
Cai and Zhang [28]	Enterprise’s leverage ratio	Debt overhang theory	An increase in leverage leads to future underinvestment, thus reducing a firm’s value.
Demir [31]	Financial and fixed investments	Portfolio theory	Rather than investing in irreversible long-term fixed investments, firms may choose to invest in reversible short-term financial investments.
De Angelo *et al*. [56]	Corporate deleveraging and financial flexibility	Debt overhang theory	Corporate deleveraging can restore adequate financial flexibility.
Zhao *et al*. [45]	Excessive capacity and innovation	Decision theory	Excessive capacity of enterprises occupies scarce resources that can be used for innovation activities, showing a crowding-out effect on innovation investment.
Ma *et al*. [11]	Decapacity and innovation	Extrusion effect theory	Decapacity policies can free up occupied resources and mitigate the crowding-out effect of innovation investment.
Kim and Kung [47]	Asset reversibility and capital allocation	Input-output analysis theory	Following an escalation in uncertainty, firms utilizing capital with lower re-deployability exhibit a greater reduction in investment.
Liu and Zhang [49]	Economic policy uncertainty and investment-financing decisions	Decision theory	Economic policy uncertainty significantly impedes real investment.

### 2.2 Research hypothesis

Throughout China’s history, many companies have experienced uncontrolled expansion and accumulated excessive debt while investing in projects. Consequently, deleveraging of these enterprises has become a key task for structural reform and sustainable development in China. Notably, highly indebted SOEs in industries characterized by overcapacity have witnessed a significant decrease in their asset-liability ratios, marking the initiation of deleveraging. For example, the growth rate of the leverage ratio in 2017 was 10.9% lower than the annual average value over the past five years. This showed dual effects: (1) it maintained control over the overall leverage ratio and accelerated the incremental growth rate of the social financing scale; (2) the year-on-year growth rate of the M2 balance decreased to 9.4% in June of the same year, indicating an optimized leverage structure. Therefore, it is essential to impose stringent control over the asset-liability ratio of SOEs burdened with overcapacity.

However, for high-growth enterprises that drive industrial structural transformation and upgrading, a flexible approach to controlling their ratio is necessary. In the past, many enterprises struggled to manage the excessive industrial expansion, causing overleveraging in their project investments. Deleveraging these enterprises has become a paramount objective of the supply-side structural reform. This article argued that in the context of deepening structural reform and providing financial services for the real economy, the reform of deleveraging policies plays a crucial role by leveraging corporate asset reversibility. Thus, the first hypothesis was proposed:

**Hypothesis 1**: A company with a higher degree of deleveraging holds more financial assets.

Currently, several industries in China, such as steel and cement, are struggling with overcapacity issues. These companies have engaged in excessive investments and inefficiently utilized extensive human resources, land and other resources. This results in high production costs within the market. In some regions, SOEs continue to pursue investment opportunities without considering the soundness of the manufacturing industry from a market-oriented perspective. Consequently, when companies borrow funds for new production capacity, they usually fail to carefully evaluate the effectiveness of these investment projects. Therefore, when external factors, such as government policies, compel companies to reduce their production capacity and eliminate excessive capacity, they are obliged to reassess their overall investment allocation and adjust their investment quotas for specific business activities. Therefore, the second hypothesis was proposed:

**Hypothesis 2**: Generally, a company that puts more efforts into reducing overcapacity holds a higher total capital investment.

Furthermore, there exists a capital misallocation, where capital is not adequately allocated between operational and investment activities. Certain business activities consume a significant amount of capital within an enterprise, but fail to generate corresponding high-profit returns. This reflects a degree of misallocation in the capital allocation structure, which can ultimately hinder sustainable development. The level of asset reversibility is correlated with asset specificity, industrial sunk costs, flows of production factors and difficulties in overcoming industry barriers. These factors collectively impact the ability of industries to transfer occupied resources to other business activities. Industries with high return on investment tend to experience overinvestment and overcapacity in specific sectors, leading to deviations from optimal capital allocation. Conversely, excessive asset reversibility may increase the proportion of financial transaction assets held by enterprises.

Typically, specialized assets face restrictions in redeployment, affecting operating activities more significantly and creating a certain level of "exit barriers" for operating capital (Wang *et al*. [42]). This aspect relates to the range of available investment options and the ease with which a company can enter or exit an industry. Enterprises with higher asset reversibility (lower asset specificity) find it easier to adjust capital allocation between physical and financial assets, reducing expenses associated with asset allocation (Benmelech [57]; Campello and Giambona [58]; Kim and Kung [47]). In general, assets with higher reversibility encounter fewer market frictions, enabling companies to sell assets rapidly in case of financial distress. Thus, this can reduce precautionary savings, improve liquidity and decrease bankruptcy risk (Caballero [59]; Bloom [60]). In addition, financial assets can be bought and sold more easily, which may crowd out investment in the real economy and lead to excessive financialization of enterprises. For instance, industries with severe overcapacity (such as steel and coal) in China exhibit weak asset reversibility. Implementing mandatory overcapacity policies can enhance capacity utilization and adjust asset structures. Finally, the third hypothesis was proposed:

**Hypothesis 3**: In an enterprise with higher corporate asset reversibility, deleveraging has more effects on the tendency of enterprises to hold financial assets, and decapacity has more effects the tendency of enterprises to hold total capital investments.

To examine the theoretical impact of asset reversibility on the relationship of deleveraging and decapacity policies with corporate capital allocation, we described the following mechanisms. (1) Low levels of asset reversibility indicate highly specific assets with low liquidation transaction prices and large losses of value upon realization. This can result in high burial costs and substantial sunk costs for investments entering or leaving the enterprise. The restricted reuse of enterprise assets increases capital deployment costs within enterprises, and the conversion of production factors becomes time-consuming. Breaking through industry barriers from external sources becomes more challenging. (2) When enterprise assets exhibit higher reversibility, the loss of value upon realization can be minimized. Operational managers can easily prioritize financial asset transactions for industrial investments, thereby enhancing the profitability of financial channels. Enterprises tend to increase the frequency of financial asset transactions. However, this can crowd out the real economy, leading to excessive financialization of enterprises.

## 3. Data and model specification

### 3.1 Data selection

The research data consisted of A-share listed companies from the Shanghai and Shenzhen Stock Exchanges from 2009 to 2019. The samples were processed as follows: (1) 2,440 observations were excluded from financial listed companies, and 2,420 observations were excluded from real estate listed companies; (2) 35,820 observations with missing data were also excluded. To mitigate the impact of outliers on the results, this paper applied winsorization to the two dependent variables (i.e., financial capital allocation and capital allocation), with a threshold of 5%. 9,506 remaining observations were obtained. The standard errors of the regression results were adjusted for enterprise clustering. (3) The industry standards came from the "Opinions of the State Council of China on Resolving Overcapacity in the Iron and Steel Industry and Realizing Growth from Difficulties" and the China Securities Regulatory Commission.

### 3.2 Variables

#### i. Corporate capital allocation

Financial capital allocation (Fca_it_) is based on the proportion of financial transaction assets of total assets:

$$Fca_{it}=FTA_{it}/TA_{it}$$ (1)

where *i* is the individual company, *t* is the time and year, *FTA* is financial transaction assets and *TA* is total assets. The larger the value, the more likely the company is to hold financial assets. Then, investment-related capital allocation (Cca_it_) is:

$$Cca_{it}=TCI_{it}/TA_{it}$$ (2)

where *TCI* is total capital investment. The larger the value, the more likely the company is to hold capital assets.

#### ii. Degree of deleveraging

Based on the study by Harford *et al*. [61], Lu *et al*. [62], and Xu *et al*. [63], the deleveraging degree (DLEV_it_) is expressed as:

$$DLEV_{it}=(LEV_{it}-LEV_{i,t-1})/LEV_{i,t-1}$$ (3)

where *LEVit* and *LEV*_*i*,*t-1*_ are the asset-liability ratio of the current and last periods, respectively. If the value is negative, then the company has deleveraged in the current period.

#### iii. Degree of decapacity

The degree of overcapacity can be measured using various methods. Two commonly used methods are TFP (e.g., Qian *et al*. [64]) and capacity utilization (e.g., Dong *et al*. [65]). The functional method of measuring productivity tends to result in misestimation, while the capacity utilization method demonstrates strong operability. This method can reflect and measure the degree of overcapacity in enterprises. Therefore, the Cobb-Douglas production function was employed to estimate the capacity utilization index of the sample enterprises based on TFP:

$$\text{ln}\;Y_{ijt}=\alpha_{0jt}+\alpha_{1jt}\;\text{ln}\;K_{ijt}+\alpha_{2jt}\;\text{ln}\;L_{ijt}+\varepsilon_{ijt}$$ (4)

where *Y* is the operating income of the listed company, *L* is the total number of employees, *K* represents the net fixed assets, *i* denotes the enterprise and *t* denotes the year. The remaining terms were calculated and recorded as the CU enterprise capacity utilization rate. *RECV* capacity reduction degree is equal to the current capacity utilization rate minus the capacity utilization rate in the previous period.

#### iv. Asset reversibility (AR)

According to Kim and Kung [47], the AR indexes are based on asset levels, industry levels, and enterprise levels. *UR*_*ajt*_ is the proportion of an asset used by the industry during the current year compared with the total consumption of the asset for the current year; *RE*_*ajt*_ is calculated using the asset-level reversibility multiplied by the number of assets used by the industry in the current year versus the number of assets consumed by the industry in the current year; *CO*_*it*_ is calculated as the fraction of the numerator (asset reversibility of the enterprise multiplied by the asset reversibility of the industry) and the denominator (an enterprise’s sales in a particular industry divided by the total sales of the enterprise for the year). The exact equation can be seen in Table 2.

**Table 2:** Definition and explanation of variables.

Variable	Definition		Variable	Calculation method
Dependent variables	Capital allocation	Financial	*Fca*	Inancial transaction assets/Total assets
		Investment-related	*Cca*	Total capital investment/Total assets
Independent variables	Deleveraging		*DLEV*	(Asset-liability ratio of the current period-asset-liability ratio of the previous period)/Asset—Liability ratio at the end of the previous period
	Decapacity		*RCEV*	(Current capacity utilization rate—Previous capacity utilization rate)/Previous capacity utilization rate
Moderating variables	Asset reversibility		*AR*	*UR*_*ajt*_ = The number of *a* assets used by *j* industry in *t* years/The total consumption of *a* assets in *t* years
				*RE*_*aj*t_ industry’s workplace reversibility = (The number of assets *a* used by industry *j* in *t* years/The number of all assets consumed by industry *j* in *t* years) × *A*_*ajt*_
				The main business of *i* enterprise in *t* years constitutes × *RE*_*ajt*_
Control variables	Enterprise growth		*Tq*	(Total stock market value + book value of debt)/ Book value of total assets
	Financial leverage		*Flr*	Total assets/Owners’ equity
	Size of enterprise		*Size*	Total liabilities (logarithm)
	Performance in the last year		*Prfrm*	Profit of the enterprise in the previous year/Total assets at the end of the previous year
	Fixed asset proportion		*Far*	Fixed assets/Total assets

#### v. Control variables

To address concerns related to omitted variables and endogeneity, control variables recommended in the literature (e.g., Gu [66]; Liu and Zhang [49]) were included, i.e., enterprise growth, financial leverage, enterprise size, previous year’s performance and the proportion of fixed assets. In addition, to calculate the leverage ratio, the median and mean values of deleveraging were used as instrumental variables, which represent the median and mean of the industry’s deleveraging degree, respectively. Table 2 provides a summary of these variables.

Based on Table 3 and Appendix 3 in S1 File, regarding the sample size, the mean, standard deviation and the maximum value of the variables, non-financial listed companies in China did not show significant differences in the proportion of total capital investment compared to financially listed companies. However, there was a slight variation in the holdings of listed companies in the sample as a proportion of their total assets. Generally, the asset reversibility index of non-financial listed companies in China generally remained consistent from 2009 to 2019.

**Table 3:** Definition and statistics of variables.

Variable	Definition		Variable	N	p25	p50	p75	Mean	Sd	Max
Dependent variables	Capital allocation	Financial	*Fca*	9506	0.093	0.158	0.263	0.201	0.147	0.585
		Investment-related	*Cca*	9506	0.302	0.458	0.615	0.456	0.209	0.818
Independent variables	Deleveraging		*DLEV*	9506	-0.079	0.006	0.104	0.016	0.195	0.468
	Decapacity		*RCEV*	9506	-0.090	0.065	0.237	0.099	0.311	0.875
Moderating variables	Asset reversibility		*AR*	9506	48.992	62.588	70.461	58.334	13.498	73.047
Control variables	Enterprise growth		*Tq*	9506	1.368	2.230	4.931	4.847	6.189	25.024
	Financial leverage		*Flr*	9506	0.000	0.606	1.413	0.939	1.040	3.698
	Size of enterprise		*Size*	9506	21.040	21.962	23.016	20.965	1.623	24.166
	Performance in the last year		*Prfrm*	9506	0.007	0.039	0.128	0.119	0.194	0.748
	Fixed asset proportion		*Far*	9506	0.139	0.252	0.396	0.278	0.168	0.615
Instrumental variables	Median of deleveraging		DLEV*	9506	-0.005	0.006	0.016	0.033	0.063	0.159
	Mean of deleveraging		*DLEV_*	9506	-0.162	0.027	0.070	0.005	0.028	0.059
	Median of decapacity		RCEV*	9506	0.112	0.249	0.915	0.087	0.085	0.236
	Mean of decapacity		*RCEV_*	9506	0.021	0.088	0.142	0.113	0.085	0.258

Data: Win.d database was obtained using the authors’ calculations.

### 3.3 Model specification

The following models were used to test Hypotheses 1 and 2 (Xu *et al*. [63]):

$$F{\text{ca}}_{it}=\alpha_{0}+\alpha_{1}DLEV_{it}+\alpha_{2}{\text{Tq}}_{it}+\alpha_{3}Flr_{\iota \tau }+\alpha_{4}Size_{it}+\alpha_{5}\text{Pr}frm_{it}+\alpha_{6}{\text{Far}}_{\text{it}}+\theta_{i}+u_{it}$$ (5)

$$C{\text{ca}}_{it}=\beta_{0}+\beta_{1}DLEV_{it}+\beta_{2}T{\text{q}}_{it}+\beta_{3}Flr_{it}+\beta_{4}Size_{it}+\beta_{5}P{\text{rfrm}}_{\text{it}}+\beta_{6}{\text{Far}}_{\text{it}}+\theta_{i}+u_{it}$$ (6)

$$C{\text{ca}}_{it}=\gamma_{0}+\gamma_{1}RCEV_{it}+\gamma_{2}Tq_{it}+\gamma_{3}Flr_{it}+\gamma_{4}Size_{it}+\gamma_{5}P{\text{rfrm}}_{\text{it}}+\gamma_{6}{\text{Far}}_{\text{it}}+\theta_{i}+u_{it}$$ (7)

$$F{\text{ca}}_{it}=\lambda_{0}+\lambda_{1}RCEV_{it}+\lambda_{2}Tq_{it}+\lambda_{3}Flr_{it}+\lambda_{4}Size_{it}+\lambda_{5}P{\text{rfrm}}_{\text{it}}+\lambda_{6}{\text{Far}}_{\text{it}}+\theta_{i}+u_{it}$$ (8)

where *i* represents the studied sample company and *t* represents the year. Corporate financial capital allocation (*Fca*) and corporate investment-related capital allocation (*Cca*) are the explained variables. The degree of deleveraging (*DLEV*) and the degree of decapacity (*RCEV*) are the core explanatory variables. The remaining variables are control variables.

**Fig 2:**
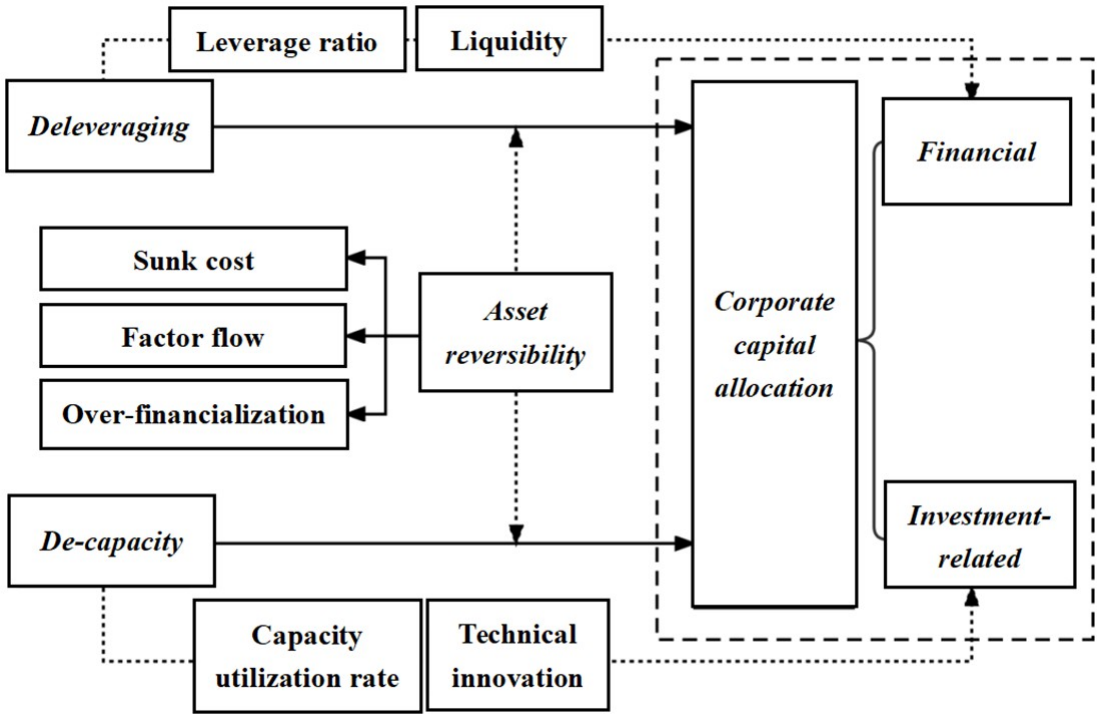


To compare and examine the effects of deleveraging and decapacity on corporate capital allocation, the indicators were first normalized. Then, the information entropy was calculated to determine the weights of the indicators for corporate capital allocation between financial and non-financial types. Thus, the weights of financial and non-financial capital allocation were 0.5695 and 0.4305, respectively. Overall, Fig 2 explains the framework of this study.

In terms of the societal benefits, this study aims to provide support for the theory that deleveraging policies can regulate enterprise leverage ratios and that decapacity policies can improve capacity utilization rates. These factors will ultimately affect the high-quality economic development.

Specifically, an appropriate leverage ratio can promote high-quality economic development by enhancing investment returns and production efficiency. It is important to note that excessively high or low financial leverage are detrimental to high-quality economic growth. Excessive corporate leverage can diminish the potential output of the economy. Characterized by instability and information asymmetry, the financial system becomes more unstable as financing scales expand. During economic downturns and declining corporate earnings, high levels of debt accumulation increase the risk of corporate solvency and bankruptcy. This can reduce the investment capacity and business performance of enterprises. Once the "debt-deflation" mechanism is triggered, the business situation and balance sheets of enterprises continue to deteriorate, resulting in a prolonged recession (Bernanke *et al*. [67]). Greater capital misallocation can hinder the quality of economic growth. Thus, to sustain healthy economic development, many countries worldwide have implemented measures to address excessive leverage.

China’s economic growth stems from technological progress driven by strong innovation capabilities. However, China’s low efficiency in capital factor utilization has led to a vicious cycle of "high investment-declining productivity-excessive capacity". By increasing the capacity utilization rate, enterprises can expand production, reduce costs, optimize capital utilization, achieve optimal output scales, increase profits and ultimately promote economic growth (Zhang and Liu [68]; Ray *et al*. [69]). Therefore, the leverage ratio and capacity utilization ratio of enterprises can significantly impact the economic growth of a productive society.

## 4. Estimation results and discussion

### 4.1 Basic model: Impact of deleveraging and decapacity on corporate capital allocation

Firstly, the effect of deleveraging and decapacity on corporate capital allocation was analyzed using fixed-effect regressions on Models (1), (2), (3), and (4), as shown in Table 4.

**Table 4:** Impact of deleveraging and decapacity on corporate capital allocation.

Variable	Fixed-effect model			
	(1)	(2)	(3)	(4)
	*Fca*	*Cca*	*Cca*	*Fca*
*DLEV*	-0.0644***	-0.0008		
	(-10.58)	(-0.57)		
*RCEV*			0.0148***	-0.0019
			(3.34)	(-0.47)
*Tq*	-0.0016*	-0.0083***	-0.0152***	-0.0018
	(-1.91)	(-6.91)	(-11.57)	(-1.57)
*Flr*	-0.0131***	-0.0928***	-0.0579***	-0.0106***
	(-4.08)	(-13.60)	(-11.34)	(-3.23)
*Size*	-0.0304***	-0.0834***	-0.1130***	-0.0333***
	(-6.46)	(-12.08)	(-16.82)	(-6.44)
*Prfrm*	0.0272*	0.0131***	-0.0030	0.0121
	(1.76)	(4.46)	(-0.15)	(0.71)
*Far*	-0.3910***	0.4460***	0.3910***	-0.3530***
	(-18.13)	(13.17)	(12.10)	(-14.91)
_cons	1.0000***	2.1190***	2.8300***	0.9250***
	(10.12)	(9.32)	(19.33)	(8.26)
Time-fixed	YES	YES	YES	YES
Individual-fixed	YES	YES	YES	YES
Time-fixed × Individual-fixed	YES	YES	YES	YES
N	9506	9506	9506	9506

Note:

***, ** and * indicate P<0.01,P<0.05 and P<0.1, respectively.

The t value corresponding to the two-sided test was output in parentheses.

Table 4 shows that the coefficients of capacity reduction variables and financial capital allocation variables were significant at the 1% level (as shown in column (1)). However, the coefficients of deleveraging and investment-related capital allocation failed to pass the significance test (as shown in column (2)). This indicates that deleveraging had a more significant promoting effect on financial capital allocation than on investment-related capital allocation. Thus, following the literature by Liu *et al*. [70], the estimated result shows that by holding other factors constant, for every 0.147 standard deviation increase in deleveraging, financial capital allocation increased by 0.947%. It should be noted that negative deleveraging variables represent stronger degrees of corporate deleveraging. Therefore, although the direction of the regression coefficient between deleveraging and financial capital allocation was negative, both variables had positive economic significance. This indicates that under stronger deleveraging policies, a company’s debt and leverage ratio would decrease at the end of the period. To avoid a financial crisis and maintain capital liquidity, companies would increase their holdings of financial assets.

The coefficients of capacity reduction variables and investment-related capital allocation variables were positive and significant at the 1% level (as shown in column (3) of Table 4). However, the coefficients of capacity reduction and financial capital allocation variables failed to pass the significance test (as shown in column (4) of Table 4). This means that under stronger decapacity policies, the capacity utilization rate would show a larger increase, and companies would allocate more funds originally intended for financial investment projects to other production-related investment projects, such as technological innovation. Therefore, more companies tend to hold total capital investments, supporting Hypotheses 1 and 2.

In general, both deleveraging and decapacity policies have significant effects on corporate capital allocation. To further compare and study the effects of these two policies, this paper introduced the following formula:

$$Z_{DLEV}=W_{1}\cdot \alpha_{1}Fca_{DLEV}+W_{1}\cdot \beta_{1}Cca_{DLEV}$$ (9)

$$Z_{RCEV}=W_{2}\cdot \gamma_{1}Cca_{RCEV}+W_{2}\cdot \lambda_{1}Fca_{RCEV}$$ (10)

where *Z*_*DLEV*_and *Z*_*RCEV*_ represent the policy effect of deleveraging and decapcity on corporate capital allocation, respectively; *W*_*1*_ and *W*_*2*_ represent the weight of corporate financial and non-financial capital allocation indicators, respectively.*ɑ*_*1*_ represents the regression coefficient of the deleveraging variable and the financial capital allocation (*Fca*) in Model (5), *β*_*1*_ represents the regression coefficient between the deleveraging variable and the capital allocation (*Cca*) in Model (6), *γ*_*1*_ represents the regression coefficient between the decapacity variable and the capital allocation (*Cca*) in Model (7) and *λ*_*1*_ represent the financial capital allocation (*Cca*) in Model (8).

Regression results in Table 4 indicate that coefficients *β*_*1*_ in column (2) and *λ*_*1*_ in column (4) failed to pass the significance test. Thus, the latter term of *Z*_*DLEV*_ and *Z*_*RCEV*_ has no practical significance. Then, we substituted the weights of corporate capital allocation indicators in Eqs. (9) and (10). *Z*_*DLEV*_ was -0.3667, showing that when all other variables remain unchanged, for every 0.147 standard deviations increase in the degree of deleveraging, the corporate capital allocation would increase by 5.39% on average. *Z*_*RCEV*_ was 0.00637, indicating that when all other variables remain unchanged, for every 0.209 standard deviations increase in the degree of decapacity, the capital allocation of the enterprise would increase by 0.133% on average.

The comparative study of the effects of deleveraging and decapacity policies on corporate capital allocation shows that the deleveraging policy has a greater impact on the financial capital allocation of enterprises, while the decapacity policy has a greater impact on investment-related capital allocation. During the period of high-quality development of the real economy, enterprises should consider high leverage, factor allocation and the continuous impact of resource flow on the real economy. Analyzing enterprises’ financial investments from the perspective of their behavioral motivation shows that enterprises increase their holdings of financial assets to hedge risks associated with fixed asset investments. During this period, enterprises are more inclined to invest in financial assets for risk mitigation. However, for enterprises with weak production technologies and low dependence on technological innovation, financial investment behavior is typically seen as speculative activities, which can increase enterprise leverage.

### 4.2 Endogenous test

#### i. Using instrumental variables

Using instrumental variables, two-stage least squares (2SLS) regression and GMM regression were conducted to solve the possible bidirectional causal issue, as shown in Table 5.

**Table 5:** Using instrument variables for endogenous test.

Variable	(1)	(2)	(3)	(4)	(5)	(6)	(7)	(8)
	*Fca_Second*	*Fca_First*	*Cca_Second*	*Cca_First*	*Cca_Second*	*Cca_First*	*Fca_Second*	*Fca_First*
*DLEV*	-0.9640***		0.0049					
	(-6.19)		(0.11)					
*RCEV*					0.0370***		-0.0309	
					(2.75)		(-1.51)	
*DLEV_iv1*		0.8040		0.2810***				
		(0.61)		(6.37)				
*DLEV_iv2*		0.4560		0.9230***				
		(0.25)		(6.46)				
*RCEV_iv1*						0.0101*		0.0053
						(1.68)		(1.29)
*RCEV_iv2*						1.052***		0.975***
						(11.87)		(22.06)
*Tq*	0.0014	0.0008*	-0.0103***	0.0007*	-0.0111***	0.0014*	-0.0009	0.0013*
	(1.37)	(1.85)	(-11.91)	(1.77)	(-9.33)	(1.84)	(-1.05)	(1.78)
*Flr cwggl*	0.0110	0.015***	-0.0865***	0.0128***	-0.0700***	0.0108***	-0.0103***	0.0086**
	(1.58)	(5.96)	(-19.78)	(5.41)	(-14.97)	(2.67)	(-3.36)	(2.18)
*Size*	0.021***	0.0058***	-0.0718***	0.0059***	-0.0848***	0.0016	-0.0262***	0.003
	(3.45)	(3.23)	(-18.46)	(3.48)	(-18.27)	(0.54)	(-7.71)	(1.02)
*Prfrm*	-0.0228	-0.0255	0.0064	-0.024**	-0.0015	0.0153	0.0186	0.0162
	(-0.91)	(-2.34)	(0.38)	(-2.23)	(-0.08)	(0.82)	(1.18)	(0.87)
*Far*	-0.390***	-0.0318**	0.299***	-0.0306**	0.370***	-0.0237	-0.327***	-0.0227
	(-13.39)	(-2.36)	(12.49)	(-2.53)	(12.83)	(-1.13)	(-16.21)	(-1.12)
_cons	0.681***	-0.0709	2.065***	-0.138***	2.303***	-0.0215	0.864***	-0.0447
	(22.76)	(-0.75)	(25.01)	(-3.91)	(23.10)	(-0.34)	(11.59)	(-0.74)
Time-fixed	YES	YES	YES	YES	YES	YES	YES	YES
Individual-fixed	YES	YES	YES	YES	YES	YES	YES	YES
Time-fixed × Individual-fixed	YES	YES	YES	YES	YES	YES	YES	YES
Kleibergen-Paap rk LM test	78.412		203.896		399.860		152.100	
	p = 0.0000		p = 0.0000		p = 0.0000		p = 0.0000	
Cragg-Donald Wald F	36.6409		114.199		265.903		102.139	
	[19.93]		[19.93]		[19.93]		[19.93]	
Sargan test	2.41886		1.5417		0.049671		2.49344	
	p = 0.1199		p = 0.2144		p = 0.8236		p = 0.1143	
N	9506	9506	9506	9506	9506	9506	9506	9506

Note:

***, ** and * indicate P<0.01, P<0.05 and P<0.1, respectively.

The t value corresponding to the two-sided test was output in parentheses. The value in square brackets shows the 10% critical value of the Stock-Yogo weak identification test.

In Table 6, the instrumental variables used in this study were the industry average (DLEV*) and the industry median (DLEV_) of the deleveraging level, which served as instruments for both the deleveraging variable and the industry average (RCEV) of the decapacity level. The average leverage ratio of a specific industry reflected the credit policy and macro debt status of that industry and was correlated with the leverage ratio of individual enterprises, thus satisfying the correlation hypothesis. Capital allocation was an internal decision made by individual enterprises and was determined by the financial situation and development strategy of that particular enterprise. Capital allocation was correlated with the average leverage ratio of the industry, thereby satisfying the exogeneity hypothesis. In addition, models were tested for non-identification, weak identification and over-identification. The p-value of the Kleibergen-Paap rk LM statistic was 0.001, and the null hypothesis that the instrumental variable was not identified was rejected at the 1% significance level. The Donald Wald F statistic was also greater than the 10% critical value of the Stock-Yogo weak identification test (13.43), indicating that the model was not subject to weak instrumental variables. Therefore, the results of instrumental variable two-stage regression were reliable.

**Table 6:** Using GMM for endogenous test.

Variable	(1)	(2)	(3)	(4)
	*Fca*	*Cca*	*Cca*	*Fca*
*DLEV*	-0.173***	-0.0070		
	(-3.12)	(-0.06)		
*RCEV*			0.0485**	-0.0103
			(2.08)	(-0.37)
*Tq*	-0.0010*	-0.0026***	-0.0018***	-0.0015**
	(-1.92)	(-6.36)	(-3.77)	(-2.21)
*Flr*	-0.0113***	-0.107***	-0.102***	-0.012***
	(-4.31)	(-42.41)	(-46.20)	(-4.81)
*Size*	-0.0228***	-0.0317***	-0.0349***	-0.033***
	(-4.66)	(-20.82)	(-23.33)	(-10.04)
*Prfrm*	0.0207*	-0.0264***	-0.0420***	0.0126
	(1.77)	(-2.75)	(-4.19)	(1.00)
*Far*	-0.382***	0.270***	0.297***	-0.347***
	(-27.19)	(25.06)	(26.35)	(-23.05)
_cons	0.649***	1.205***	1.262***	0.664***
	(22.57)	(37.74)	(40.35)	(21.08)
Time-fixed	YES	YES	YES	YES
Individual-fixed	YES	YES	YES	YES
Time-fixed × Individual-fixed	YES	YES	YES	YES
N	9506	9506	9506	9506

Note:

***, ** and * indicate P<0.01, P<0.05 and P<0.1, respectively.

The t value corresponding to the two-sided test was output in parentheses.

Following the resolution of the bidirectional causality issue in the model, the estimation results of IV-2SLS in columns (1) and (5) of Table 5 revealed significant findings. At the 5% significance level, the coefficient of the deleveraging variable was significantly negative, indicating that corporate deleveraging had a substantial impact on the company’s financial assets. However, the coefficient of decapacity was significantly positive, suggesting that increasing decapacity tended to increase the company’s holdings of capital assets. However, columns (3) and (7) of Table 5 demonstrate that the coefficient of deleveraging on capital assets and the coefficient of decapacity on financial capital were insignificant. This suggests that leverage had a more adverse effect on the propensity to hold financial assets, whereas corporate decapacity had a more adverse effect on the tendency to hold capital assets.

Typically, instrumental variables are tested using 2SLS, which is the most efficient method under the assumption of spherical disturbances. However, under the assumption of heteroscedasticity or autocorrelation, GMM estimation is more efficient. Therefore, in this study, both 2SLS and GMM were employed as the instrumental variable test methods. Both methods confirmed the reliability of the basic model. The analysis also indicates that Models (5) and (7) were more suitable for investigating the impact of deleveraging and decapacity on corporate capital allocation.

#### ii. Using propensity score matching for endogenous test

**Fig 3:**
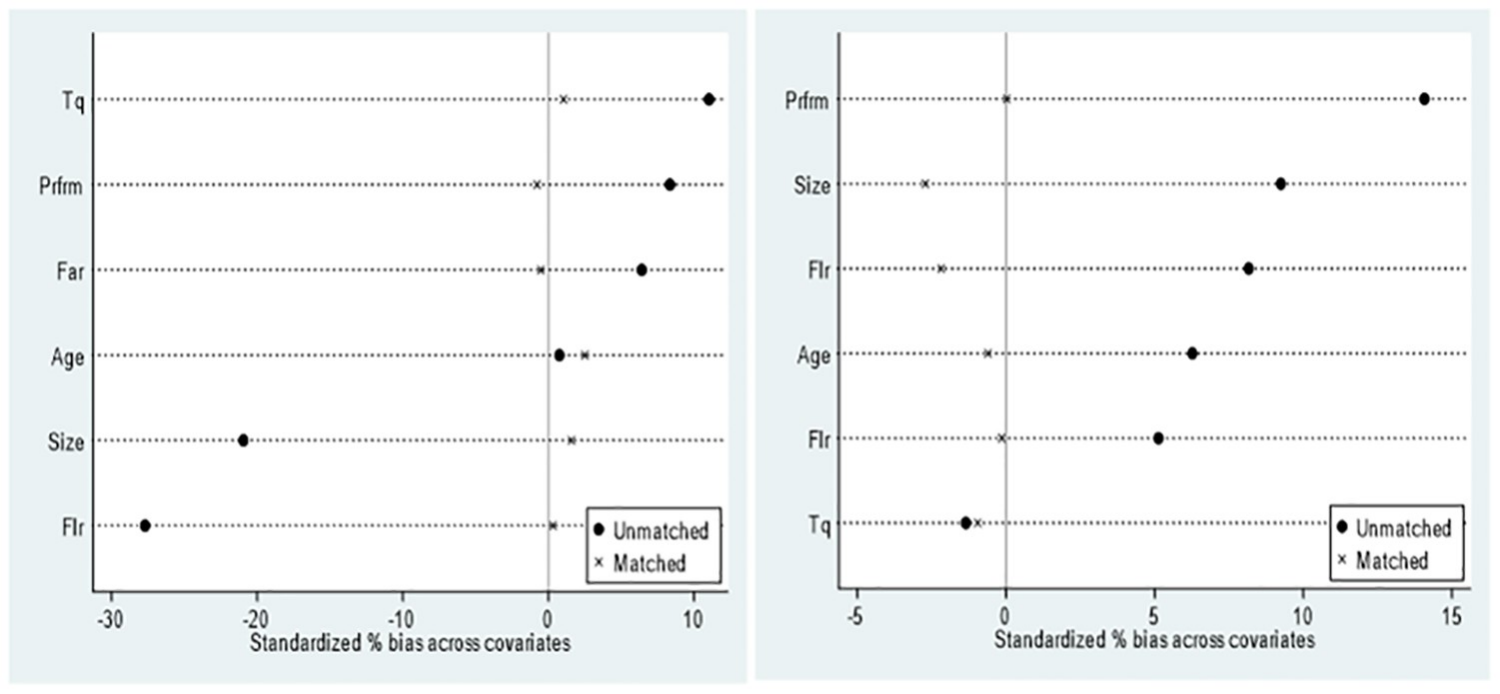


Given the possibility of systematic differences between companies that engage in deleveraging and decapacity behaviors and those that do not, sample self-selection bias may influence the relationship between supply-side structural reform and corporate capital allocation. To address this concern, this study employed the PSM model to examine the net effect of deleveraging policies and decapacity policies on corporate capital allocation. To match and filter samples without deleveraging and with decapacity, the explanatory variable by Xu *et al*. (2020) [63] was utilized. Control variables that affect *Treat_One* were selected as matching variables. These control variables included enterprise growth (*Tq*), financial leverage ratio (*Flr*), corporate scale (*Size*), last year’s operating conditions (*Prfrm*), fixed assets ratio (*Far*) and listing age (*Age*). Subsequently, logit regression was used to estimate the propensity scores of enterprises with deleveraging and decapacity, and those without. Nearest neighbor matching, radius matching and kernel matching techniques were employed to match the estimated propensity scores. After PSM, a total of 9,506 samples were obtained in the deleveraging on financial asset allocation sample, with 4,499 and 5,007samples in the treatment and control groups, respectively. For the decapacity sample, 4,508 samples were obtained, with 1,319 and 3,189 samples in the treatment and control groups, respectively. The results are presented in Table 7, and the standard deviation of variables is shown in Fig 3.

**Table 7:** Using propensity score matching for endogenous test.

Variable	Unmatched- U/ Matched- M	Treatment group	Control group	Discrepancy (%)	Discrepancy narrow-down	T	P
*Tq*	U	4.718	4.092	11.0	90.5	5.40	0.000
	M	4.714	4.654	1.0		0.48	0.633
	U	4.634	3.859	14.1	95.2	6.23	0.000
	M	4.636	4.599	0.7		0.36	0.719
*Flr*	U	1.087	1.369	-27.7	98.8	-13.4	0.000
	M	1.087	1.084	0.3		0.16	0.870
	U	1.158	1.109	5.1	97.1	1.40	0.162
	M	1.158	1.159	-0.2		-0.05	0.959
*Size*	U	21.08	21.428	-21.0	92.4	-10.2	0.000
	M	21.08	21.054	1.6		92.4	0.449
	U	21.242	21.308	-4.0	30.7	-1.80	0.071
	M	21.243	21.289	-2.8		-1.55	0.120
*Prfrm*	U	0.127	0.111	8.4	90.7	4.08	0.000
	M	0.127	0.129	-0.8		-0.35	0.723
	U	19.166	18.921	14.1	99.9	3.83	0.000
	M	19.166	19.166	0.0		0.00	0.996
*Far*	U	0.282	0.272	6.4	92.1	3.13	0.002
	M	0.282	0.283	-0.5		-0.24	0.811
	U	0.276	0.277	-0.5	83.2	-0.23	0.822
	M	0.276	0.277	-0.1		-0.05	0.962
*Age*	U	16.748	16.703	0.8	-226.9	0.37	0.709
	M	16.747	16.600	2.5		1.19	0.235
	U	16.555	16.187	6.3	90.3	1.71	0.087
	M	16.555	16.591	-0.6		-0.21	0.836

Data: The above results were obtained using Stata.

Table 7 presents the balance test results of PSM. The majority of variables exhibited significance at the 5% level, and the t-test for the difference between the treatment group and the control group after matching was insignificant. Therefore, the selection of matching variables and methods was appropriate, and the matching estimates were accurate.

Fig 3 also illustrates that after PSM, the sample deviation decreased compared to that before the matching, and the distribution of the samples became more concentrated. Utilizing the matched samples, we retested the estimation results of the impact of the deleveraging policy on corporate financial assets and the impact of the decapacity policy on corporate capital assets, as presented in Table 8. The regression results were consistent with those estimated by the benchmark model in Table 4.

**Table 8:** PSM estimation results.

Variable		Unmatched- U/ Matched- M		Treatment group	Control group	ATT	Discrepancy (%)	T
Deleveraging on financial asset	*Fca*	Unmatched		-1.807	-1.897	0.091	0.015	5.95***
		Matched	Nearest neighbor	-1.807	-1.865	0.058	0.021	2.77***
			Radius matching	-1.807	-1.863	0.057	0.015	3.63***
			Kernel matching	-1.807	-1.863	0.056	0.016	3.5***
Decapacity on non-financial capital asset	*Cca*	Unmatched		0.465	0.473	-0.011	0.005	-2.27***
		Matched	Nearest neighbor	0.467	0.482	-0.016	0.007	-2.46***
			Radius matching	0.468	0.459	0.009	0.005	2.05***
			Kernel matching	0.468	0.456	0.012	0.005	2.53***

Data: The above results were obtained using Stata.

Table 8 presents the average treatment effect (ATT) values before and after employing the three different sample matching methods. By accounting for the non-randomness of deleveraging and decapacity, all three matching methods reveal that the ATT of deleveraging on corporate financial assets wass statistically significant at the 1% significance level. Similarly, decapacity exhibited a significant effect on corporate capital assets, with the ATT being significant at the 1% level. These findings indicate that deleveraging and decapacity had distinct effects on corporate asset allocation.

#### iii. Difference in Difference (DID)

Finally, a double-difference test model was constructed (Table 9) using the matched and unmatched samples using the PSM model (columns (5) and (6) of Table 9) to identify the causal relationship between deleveraging, decapacity and corporate capital allocation.

**Table 9:** DID for impact before and after structural reform in 2015.

Variable	DID				PSM+DID	
	(1)	(2)	(3)	(4)	(5)	(6)
	*Fca*	*Cca*	*Cca*	*Fca*	*Fca*	*Fca*
*DLEV_Treat×Post*	-0.0209***	0.0057			-0.0208***	
Deleverage after 2015	(-5.15)	(1.47)			(-5.00)	
*RCEV_Treat×Post*			0.0117**	0.0006		0.0145**
Decapacity after 2015			(1.98)	(0.12)		(2.33)
*Tq*	-0.0012**	-0.0144***	-0.0144***	-0.0019**	-0.002***	-0.0164***
	(-2.27)	(-14.57)	(-14.56)	(-2.24)	(-5.44)	(-32.49)
*Flr*	-0.0138***	-0.0738***	-0.0739***	-0.0147***	-0.0137***	-0.0025***
	(-4.24)	(-15.06)	(-15.17)	(-4.54)	(-7.40)	(-10.21)
*Size*	-0.0343***	-0.104***	-0.105***	-0.0352***	-0.0344***	-0.141***
	(-7.27)	(-18.68)	(-18.83)	(-7.52)	(-15.92)	(-55.76)
*Prfrm*	0.0303*	0.0124	0.0115	0.0307*	0.0299***	0.0297***
	(1.93)	(0.66)	(0.61)	(1.95)	(3.52)	(16.67)
*Far*	-0.393***	0.322***	0.322***	-0.391***	-0.394***	0.323***
	(-17.91)	(11.46)	(11.45)	(-17.80)	(-33.71)	(19.68)
*_cons*	1.067***	2.700***	2.711***	1.094***	1.068***	2.937***
	(10.72)	(19.07)	(19.18)	(10.93)	(13.30)	(49.68)
Time-fixed	YES	YES	YES	YES	YES	YES
Individual-fixed	YES	YES	YES	YES	YES	YES
Time-fixed*×* Individual-fixed	YES	YES	YES	YES	YES	YES
PSM	NO	NO	NO	NO	YES	YES
N	9506	9506	9507	9507	3634	3634

Data: The above results were obtained using Stata.

***, ** and * indicate P<0.01, P<0.05 and P<0.1, respectively.

The t value corresponding to the two-sided test was output in parentheses.

The underutilized PSM model (columns 1~4 of Table 8) was based on reducing negative bias due to missing variables. In the study of the deleveraging policy effect, we set the treatment group as the enterprise with deleveraging behavior (DLEV_Treat = 1) and set the control group as the enterprise without deleveraging behavior (DLEV_Treat = 0). In the study of the decapacity policy effect, we set the treatment group as the enterprise with deleveraging behavior (RCEV_Treat = 1) and set the control group as the enterprise without the decapacity behavior (RCEV_Treat = 0). The supply-side structural reform was first formally proposed at the end of 2015. Thus, the sample before the supply-side structural reform (2015 and before) was set as Post = 0; after the supply-side structural reform (2015 and before), the sample was set as Post = 1. Then, the impact of the interaction term DLEV_Treat×Post on the financial capital allocation of enterprises and the impact of RCEV_Treat×Post on the capital allocation of enterprises were tested.

The first four columns of Table 9 indicate that the samples after PSM were not selected for estimation. The coefficient of DLEV_Treat×Post for financial assets was significantly negative at the 1% level, but there was no significant difference for capital assets. This shows that with a higher degree of deleveraging, the company would hold more financial assets following the implementation of the supply-side structural reform policy. The coefficient of RCEV_Treat×Post was significantly positive at the 10% level; however, it did not have a significant impact on financial assets. This indicates that after the implementation of the supply-side structural reform policy, at a higher degree of decapacity, companies tend to increase their investments in capital assets. Columns (5) and (6) of Table 9 present results for the sample estimation of double differencing after selecting PSM. DREV_Treat×Post and RCEV_Treat×Post were significant at 1% and 5%, respectively, and the sign direction of the coefficients were consistent with the previous estimation results. The analysis results still supported Hypothesis 1 in this paper, suggesting that the conclusion was valid even after considering the endogeneity in the model.

The underlying assumption of the DID method is that the treatment and control groups satisfy the parallel trend assumption. Therefore, the dynamic effects of the deleveraging and decapacity were tested using the event study approach. The previous period of policy implementation was taken as the base period. Appendix 4 in S1 File shows the estimation results of the deleveraging and decapacity on corporate financial capital allocation and investment-related capital allocation at 95% confidence level, respectively. It can be seen that the estimated coefficients of *DLEV_Treat×Post* and *RCEV_Treat×Post* for the 14 years prior to policy implementation were insignificant. This indicates that there was no significant difference between the treatment and control groups. Thus, the parallel trend hypothesis held. After policy implementation, their estimated coefficients were significantly negative and positive at the 5% level, respectively, indicating the above regression results were robust.

To further verify that the results were not caused by unobservable company-level factors, we conducted a placebo test by randomly assigning enterprises to the treatment group. Specifically, enterprises were randomly selected from the samples as the treatment group, and the remaining enterprises were the control group. The control variables for the placebo test were the same as the above. If the above regression results are reasonable, the coefficients of *DREV_Treat×Post* and *RCEV_Treat×Post* should be insignificant overall in the placebo test with 1000 randomly assigned shocks. The estimation results are shown in Appendix 5 in S1 File.

### 4.3 Robustness test

To enhance the robustness of the main regression findings, this study employed three methods to re-validate the main regression: incorporating a one-period lag for the decapacity and deleveraging variables, adjusting control variables and reducing the time sample.

#### i. Incorporating a one-period lag of explanatory variables

Different types of businesses exhibit varying leverage ratios. The China State Council has recognized that high-tech, bio-pharmaceutical and other companies may require and sustain high leverage due to their substantial initial investment capital and relatively delayed returns. In the context of China’s supply-side structural reforms, structural deleveraging is not universally applicable but should be gradually implemented for non-financial firms. Considering the time for the deleveraging policy to take effect within the target industry and the availability of accurate data on the intensity of enterprise deleveraging, there is a significant lag during this period. Therefore, this paper examined the relationship between the intensity of deleveraging and decapacity with a one-period lag to test the robustness of the main regression. In addition, this method can also assess the causal relationship between the strength of deleveraging and the propensity of enterprises to hold financial assets, as well as the relationship between the strength of decapacity and the total capital investments by enterprises.

#### ii. Modification of control variables

The control variables were adjusted to test their sensitivity, as shown in columns (3) and (4) of Table 10. By excluding the control variable for enterprise growth, it is verified that the degree of deleveraging and decapacity can significantly influence the asset holdings of enterprises. In summary, when the control variable for enterprise growth was removed from Models (1) and (3), a higher degree of deleveraging was associated with increased financial asset holdings by the company. Similarly, a higher degree of decapacity was associated with increased capital asset holdings by the company. Hence, the conclusion remains consistent with the previous findings.

**Table 10:** Results of the robustness test.

Variable	One period lag of explanatory variable		Modification of control variables		Narrow down the time frame			
					Before 2015		After 2015	
	(1)	(2)	(3)	(4)	(5)	(6)	(7)	(8)
	*Fca*	*Cca*	*Fca*	*Cca*	*Fca*	*Cca*	*Fca*	*Cca*
*L_DLEV*	-0.0400***							
	(-7.07)							
*L_RCEV*		0.0108***						
		(2.60)						
*DLEV*			-0.0658***		-0.0001		-0.0156*	
			(-10.66)		(-0.05)		(-1.75)	
*RCEV*				0.0181***		0.0027		0.0221***
				(3.63)		(1.32)		(3.46)
*Tq*	-0.0018*	-0.0161***			-0.003**	-0.0154***	0.0058*	-0.0395***
	(-1.79)	(-11.38)			(-2.29)	(-9.03)	(1.79)	(-7.44)
*Flr*	-0.0121***	-0.0575***	-0.0153***	-0.0744***	-0.0037	-0.0596***	0.0058*	-0.0395***
	(-3.51)	(-11.23)	(-4.87)	(-12.96)	(-0.85)	(-7.38)	(1.79)	(-7.44)
*Size*	-0.0364***	-0.111***	-0.0248***	-0.0652***	-0.04***	-0.0845***	-0.00001**	-0.129***
	(-7.21)	(-15.68)	(-6.08)	(-11.12)	(-4.68)	(-8.06)	(-2.18)	(-12.33)
*Prfrm*	0.0221	-0.0092	0.0233	-0.016	-0.0294	0.0582**	0.0194	0.005
	(1.36)	(-0.45)	(1.51)	(-0.78)	(-1.20)	(2.01)	(1.30)	(0.27)
*Far*	-0.392***	0.402***	-0.395***	0.360***	-0.320***	0.484***	-0.285***	0.410***
	(-17.05)	(12.06)	(-18.29)	(10.26)	(-10.34)	(9.79)	(-9.22)	(8.42)
_cons	1.109***	2.871***	0.856***	1.863***	1.07***	2.233***	0.244***	3.279***
	(10.02)	(18.4)	(9.67)	(14.56)	(6.37)	(10.23)	(12.80)	(14.19)
Time-fixed	YES	YES	YES	YES	YES	YES	YES	YES
Individual-fixed	YES	YES	YES	YES	YES	YES	YES	YES
Time-fixed×Individual-fixed	YES	YES	YES	YES	YES	YES	YES	YES
N	8745	8745	7604	7604	2994	2994	3951	3951

Data: The results were calculated using Stata.

***, ** and * indicate P<0.01, P<0.05, and P<0.1, respectively.

The t value corresponding to the two-sided test was output in parentheses.

#### iii. Narrowing down the time frame

To further investigate the robustness of the results, the time sample was narrowed down by dividing it into two periods: before and after the introduction of supply-side structural reforms in 2015. As shown in columns (5) and (7) of Table 10, the coefficient of the deleveraging variable never became significantly negative at the 5% significance level. This indicates that after the implementation of the supply-side structural reform policy, the intensity of deleveraging increased, significantly enhancing financial capital allocation by enterprises. Prior to 2015, the coefficient of the decapacity variable was significant at the 10% significance level. This is because, before the formal proposal of the supply-side structural reform policy, the government had intervened multiple times to reduce overcapacity in various industries. However, the impact of such measures on reducing overcapacity was not pronounced. After 2015, the coefficient of decapacity significantly increased, indicating a more noticeable effect of decapacity on the capital allocation of enterprises. These findings aligned with the estimation results of the benchmark model presented in this study. The overall results of the robustness test are presented in Table 10.

### 4.4 Test of mechanisms: Results considering corporate asset reversibility

To further examine Hypothesis 3, this section analyzed the relationship between deleveraging policies, decapacity policies and corporate capital allocation from the perspective of corporate asset reversibility. According to the input-output table data for 2018, a reversibility index of the enterprise was constructed. Then, asset reversibility (AR) and interactions between asset reversibility and the deleveraging policy (AR*DLEV) were added to Eqs. (5) and (6). Asset reversibility (AR) and interactions between asset reversibility and the decapacity policy (AR*RCEV) were added to Eqs. (7) and (8). The new models are the followings:

Fcait=β0+β1DLEVit+β2ARit*DLEVit+β3ARit+β4Tqit+β5Flrit+β6Sizeit+β7Prfrmit+β8Farit+θ+iuit (11)

$$\begin{array}{c} C{\text{ca}}_{it}=\beta_{0}+\beta_{1}DLEV_{it}+\beta_{2}AR_{it}*DLEV_{it}+\beta_{3}AR_{it}+\beta_{4}T{\text{q}}_{it}+\beta_{5}Flr_{it}\\ +\beta_{6}Size_{it}+\beta_{7}P{\text{rfrm}}_{\text{it}}+\beta_{8}{\text{Far}}_{\text{it}}+\theta_{i}+u_{it} \end{array}$$ (12)

$$\begin{array}{c} C{\text{ca}}_{it}=\gamma_{0}+\gamma_{1}RCEV_{it}+\gamma_{2}AR_{it}*RCEV_{it}+\gamma_{3}AR_{it}+\gamma_{4}Tq_{it}+\gamma_{5}Flr_{it}\\ +\gamma_{6}Size_{it}+\gamma_{7}P\text{rfrm}+\gamma_{8}{\text{Far}}_{\text{it}}+\theta_{i}+u_{it} \end{array}$$ (13)

$$\begin{array}{c} F{\text{ca}}_{it}=\lambda_{0}+\lambda_{1}RCEV_{it}+\lambda_{2}AR_{it}*RCEV_{it}+\lambda_{3}AR_{it}+\lambda_{4}Tq_{it}+\lambda_{5}Flr_{it}\\ +\lambda_{6}Size_{it}+\lambda_{7}P\text{rfrm}+\lambda_{8}{\text{Far}}_{\text{it}}+\theta_{i}+u_{it} \end{array}$$ (14)

Then, fixed-effect regression and GMM regression on models were estimated, respectively. The results are shown in Table 11.

**Table 11:** Analysis of the moderating effect of corporate asset reversibility.

Variable	FE				GMM			
	(1)	(2)	(3)	(4)	(5)	(6)	(7)	(80
	*Fca*	*Cca*	*Cca*	*Fca*	*Fca*	*Cca*	*Cca*	*Fca*
*DLEV*	-0.121***	-0.0529			-5.484**	0.401		
	(-3.70)	(-1.41)			(-2.50)	(0.20)		
AR*DLEV	0.001*	-0.00038			0.0899**	-0.0083		
	(1.89)	(-0.60)			(2.47)	(-0.25)		
*RCEV*			0.0151***	-0.0007			0.0502**	-0.0656
			(3.23)	(-0.18)			(2.19)	(-1.57)
AR*RCEV			0.000002***	-0.00000004			0.000002***	-0.0000004
			(9.51)	(-0.14)			(4.56)	(-0.35)
*AR*	0.0005**	0.0007**	0.0005	0.0003	-0.0011	0.0010*	0.0007***	0.0003**
	(2.30)	(2.52)	(1.62)	(1.31)	(-1.50)	(1.67)	(5.51)	(2.14)
*Tq*	-0.001	-0.0141***	-0.0149***	-0.0017	0.0008	-0.0011**	-0.0017***	0.0001
	(-1.08)	(-12.23)	(-11.42)	(-1.60)	(0.89)	(-2.13)	(-3.62)	(0.12)
*Flr*	-0.0131***	-0.0654***	-0.0597***	-0.0103***	0.0012	-0.109***	-0.102***	-0.0043*
	(-4.14)	(-13.64)	(-11.76)	(-3.10)	(0.21)	(-28.97)	(-45.72)	(-1.95)
*Size*	-0.028***	-0.101***	-0.112***	-0.0324***	-0.0146***	-0.0278***	-0.0346***	-0.0184***
	(-5.82)	(-16.39)	(-16.67)	(-6.41)	(-3.48)	(-10.64)	(-22.71)	(-12.38)
*Prfrm*	0.0191	0.0092	-0.0066	0.0103	0.011	-0.0313***	-0.0386***	0.0287***
	(1.14)	(0.49)	(-0.33)	(0.60)	(0.54)	(-3.21)	(-3.85)	(2.79)
*Far*	-0.375***	0.347***	0.393***	-0.351***	-0.299***	0.321***	0.301***	-0.261***
	(-16.46)	(11.99)	(12.21)	(-14.67)	(-13.78)	(19.75)	(26.38)	(-28.17)
*_cons*	0.990***	2.682***	2.782***	0.898***	0.644***	1.095***	1.213***	0.650***
	(9.36)	(19.28)	(18.87)	(8.17)	(8.12)	(29.06)	(35.73)	(19.65)
Time-fixed	YES	YES	YES	YES	YES	YES	YES	YES
Individual-fixed	YES	YES	YES	YES	YES	YES	YES	YES
Time-fixed×Individual-fixed	YES	YES	YES	YES	YES	YES	YES	YES
N	7407	7407	7407	7407	7407	7407	7407	7407

Data: The above results were calculated using Stata.

***, ** and * indicate P<0.01, P<0.05, and P<0.1, respectively.

The t value corresponding to the two-sided test was output in parentheses.

Under the fixed effect model, in the financial capital allocation equation, the interaction term between asset reversibility and deleveraging passed the 10% significance test (column (1) of Table 11). However, the interaction term and the coefficient of the asset did not pass the significance test (column (2) of Table 11). All interaction terms between asset reversibility and decapacity passed the 1% significance test (column (3) of Table 11), whereas the coefficient of the interaction terms and financial assets did not pass the significance test (column (4) of Table 11). The GMM estimation results aligned with the fixed effects in terms of coefficient direction and significance.

The deleveraging variable had an opposite sign to the coefficient of the interaction term in this equation, indicating that enhancing corporate asset reversibility can reduce the impact of deleveraging policies on corporate financial capital. This suggests that when corporate assets are highly reversible, their liquidity improves, leading to optimal liquidation transaction prices and reduced probabilities of financial crises. Moreover, under the same-level deleveraging policy, the increase in financial asset investment becomes smaller, thereby diminishing the impact of deleveraging on corporate financial capital allocation. Conversely, in the non-financial capital equation, both the decapacity variable and its interaction term coefficient were positive. This indicates that corporate asset reversibility can positively influence the relationship between the decapacity policy and the capital allocation of enterprises. These findings support the theoretical transmission mechanism analysis of asset reversibility and confirm Hypothesis 3.

In summary, the results demonstrate that corporate asset reversibility can positively affect the relationship between the decapacity policy and corporate capital allocation. This finding aligns with the theoretical transmission mechanism analysis discussed earlier and confirms the validity of Hypothesis 3.

## 5. Homogeneity analysis

An essential objective of the deleveraging policy is to reduce overcapacity. Corporate capital allocation relies not only on internal capital and policy effects but also on factors, such as geographical location, factor intensity, and financial factors. These factors can have asymmetric effects on heterogeneous enterprises. In this study, the asset reversibility index of the sample companies was utilized and classified based on region and factor density. The descriptive results were summarized in the attached table. Does regional economic development impact the acquisition of assets by local enterprises? Economically developed areas generally possess abundant resources, have higher asset availability and exhibit greater optimism regarding the liquidation value of corporate assets. Additionally, differences in industry endowments may also influence corporate asset levels.

### 5.1 Differences between regional enterprises

To investigate the potential variations in the moderating effect of asset reversibility among listed companies in different regions, this study categorized the listed companies into three groups based on their geographical location: eastern, central and western regions. The analysis included three main regressions. The suest estimation was employed to assess the samples, followed by an inter-group coefficient difference test. The three primary regression results are presented in Table 12.

**Table 12:** Heterogeneity of corporate asset reversibility in different regions of China.

Variable	East		Central		West	
	(1)	(2)	(3)	(4)	(5)	(6)
	*Fca*	*Cca*	*Fca*	*Cca*	*Fca*	*Cca*
*DLEV*	-4.944**		-3.937		-0.639	
	(-2.20)		(-1.05)		(-0.13)	
AR*DLEV	0.0807**		0.064		0.010	
	(2.16)		(1.03)		(0.12)	
*RCEV*		0.0166**		0.0232*		0.027*
		(2.36)		(1.70)		(1.83)
AR*RCEV		-0.0000009		0.000003**		0.000005
		(-0.18)		(2.22)		(0.64)
*AR*	-0.0009	0.001***	-0.0006	0.0007**	-0.0007	0.0011***
	(-1.19)	(6.84)	(-0.56)	(2.41)	(-0.52)	(3.17)
*Tq*	0.0004	-0.0006	-0.0006	-0.0003	0.0041**	-0.0053***
	(0.43)	(-1.29)	(-0.29)	(-0.41)	(2.05)	(-4.47)
*Flr*	-0.0024	-0.116***	-0.0009	-0.0967***	-0.0084	-0.0799***
	(-0.53)	(-44.06)	(-0.13)	(-21.57)	(-0.68)	(-15.14)
*Size*	-0.0145***	-0.0339***	-0.0200***	-0.0256***	-0.0167***	-0.0456***
	(-3.99)	(-19.10)	(-4.20)	(-7.12)	(-4.37)	(-11.04)
*Prfrm*	0.0031	-0.0221**	0.0994***	-0.0715***	-0.008	-0.0404*
	(0.16)	(-1.99)	(3.72)	(-3.32)	(-0.36)	(-1.67)
*Far*	-0.303***	0.280***	-0.199***	0.240***	-0.263***	0.311***
	(-13.71)	(20.87)	(-7.23)	(8.66)	(-11.51)	(10.60)
*_cons*	0.639***	1.350***	0.705***	1.250***	0.673***	1.345***
	(9.16)	(9.84)	(6.95)	(7.20)	(8.06)	(7.19)
Time-fixed	YES	YES	YES	YES	YES	YES
Individual-fixed	YES	YES	YES	YES	YES	YES
Time-fixed×Individual-fixed	YES	YES	YES	YES	YES	YES
N	4677	4677	1464	1464	1266	1266
chi2(1)	29.31	1.01	0.00	24.87	0.13	0.06
P	0.000	0.3161	0.954	0.000	0.721	0.808

Data: The above results were calculated using Stata.

***, ** and * indicate P<0.01, P<0.05 and P<0.1, respectively.

The t value corresponding to the two-sided test was output in parentheses.

The interaction coefficients reveal that the interaction term between deleveraging and asset reversibility in the eastern region, as well as the interaction term between decapacityand asset reversibility in the central region, were statistically significant. However, the western region did not exhibit significant results. This suggests that the asset reversibility of listed companies in the eastern region negatively moderated the impact of deleveraging policies, while that in the central region mitigated the influence of decapacity policies on capital allocation. This phenomenon can be attributed to the superior transportation infrastructure in the east, resulting in lower transaction costs for enterprises and facilitating investment transactions. In contrast, the central region can benefit from abundant natural resources. Consequently, companies in these two regions find it easier to possess highly liquid assets, leading to more frequent asset settlement transactions. Implementing deleveraging and decapacity policies has significantly changed the financial transaction assets of listed companies. The moderating effect of reversibility on capital allocation has been more pronounced in these two regions.

### 5.2 Difference in enterprise feature intensity

The production factor not only reflects the capacity utilization rate of enterprises, but also has a correlation with asset reversibility. To examine the impact of asset reversibility on corporate capital allocation across different production factor intensities and to explore potential heterogeneity in the transmission mechanism, the sample companies were classified into four industry types based on the "Guidelines for Industry Classification of Listed Companies" published by the China Securities Regulatory Commission in 2021. These categories included labor-intensive, capital-intensive, technology-intensive and resource-intensive industries. Furthermore, the suest estimation was conducted on the samples, and an inter-group coefficient difference test was performed. The GMM estimation results using instrumental variables are presented in Table 13.

**Table 13:** Estimation results based on industry types.

Variable	Labor-intensive		Capital-intensive		Technology-intensive		Resource-intensive	
	(1)	(2)	(3)	(4)	(5)	(6)	(7)	(8)
	*Fca*	*Cca*	*Fca*	*Cca*	*Fca*	*Cca*	*Fca*	*Cca*
*DLEV*	0.546		6.231		-3.143***		-8.066	
	(0.13)		(1.04)		(-3.03)		(-0.39)	
AR*DLEV	-0.0100		-0.109		0.0497***		0.154	
	(-0.13)		(-1.04)		(2.95)		(0.39)	
*RCEV*		0.0296		0.0362**		0.0362**		0.0270
		(0.93)		(2.33)		(2.33)		(0.46)
AR*RCEV		-0.0001		-0.000005		0.000003***		-0.0003
		(-1.23)		(-0.77)		(2.72)		(-0.28)
*AR*	0.0007	0.0009	-0.00003	0.0007*	-0.0006	0.0009***	-0.0089	0.0038
	(1.05)	(1.36)	(-0.02)	(1.70)	(-1.31)	(5.88)	(-0.34)	(1.00)
*Tq*	0.0221***	0.0127***	-0.0025	-0.0016*	0.0012**	-0.0003	-0.0033	0.0075**
	(3.09)	(2.93)	(-0.94)	(-1.67)	(2.19)	(-0.74)	(-0.24)	(2.16)
*Flr*	-0.0461***	-0.141***	-0.0108	-0.112***	0.0035	-0.116***	-0.0438	-0.109**
	(-5.12)	(-14.65)	(-0.84)	(-25.36)	(0.90)	(-42.62)	(-0.50)	(-2.28)
*Size*	-0.0223*	-0.0163**	-0.0266***	-0.0372***	-0.0182***	-0.0381***	-0.0393	-0.0006
	(-1.76)	(-2.01)	(-2.73)	(-10.42)	(-7.17)	(-20.12)	(-0.98)	(-0.01)
*Prfrm*	-0.0639	-0.0247	0.0158	-0.0551**	0.0416**	-0.0288**	0.0524	0.0429
	(-1.50)	(-0.88)	(0.30)	(-2.26)	(2.56)	(-2.52)	(0.44)	(0.42)
*Far*	-0.310**	0.367***	-0.197**	0.358***	-0.310***	0.174***	0.250	0.258
	(-2.35)	(7.43)	(-2.00)	(11.21)	(-16.92)	(12.61)	(0.40)	(1.02)
*_cons*	0.761**	0.867***	0.862***	1.403***	0.690***	1.144***	1.451*	0.613
	(2.51)	(4.01)	(4.12)	(9.30)	(13.23)	(8.32)	(1.82)	(0.69)
Time-fixed	YES	YES	YES	YES	YES	YES	YES	YES
Individual-fixed	YES	YES	YES	YES	YES	YES	YES	YES
Time-fixed×Individual-fixed	YES	YES	YES	YES	YES	YES	YES	YES
N	480	438	1092	953	4968	4385	134	131
chi2(1)	0.22	0.16	0.05	1.30	53.77	61.71	2.37	1.13
P-value	0.637	0.6873	0.832	0.254	0.000	0.000	0.124	0.287

Data: The above results were calculated using Stata.

***, ** and * indicate P<0.01, P<0.05 and P<0.1, respectively.

The t value corresponding to the two-sided test was output in parentheses.

In terms of financial capital allocation, the interaction coefficients in columns (1), (3) and (7) of Table 13 did not pass the significance test. However, for the deleveraging variable and the asset reversibility of technology-intensive enterprises in column (5), the coefficient was significant at the 5% level. This indicates that reversibility plays an inhibitory role in the relationship between deleveraging policies and financial assets. Similarly, in the equation for non-financial capital allocation, only the interaction coefficient between decapacity and asset reversibility variables of technology-intensive enterprises in column (6) passed the significance test at the 10% level. Compared to the deleveraging policy, asset reversibility can strengthen the impact of the decapacity policy, leading enterprises to prefer acquiring capital assets. Technology-intensive enterprises are characterized by cutting-edge technologies, high automation, rapid obsolescence and substantial funding requirements for research and development. They are expected to reduce their holdings of financial assets and increase investment in technology-related innovative production inputs.

These results suggest that considering the regional and industrial heterogeneity of listed companies, their asset reversibility in different regions will have varying impacts on the relationships between deleveraging policies, decapacity policies and corporate capital allocation.

Furthermore, the asset reversibility of listed companies in the eastern and central regions was higher during the implementation of the deleveraging policy (as shown in Appendix 2 in S1 File). Appendix 2 in S1 File presents the statistical results of the regions where the enterprises are located, indicating that the eastern and central regions have higher asset reversibility scores. For instance, the median values of this indicator in Tianjin, Guangdong and Zhejiang were 66.97, 66.79, and 64.78, respectively. Listed companies in western regions had lower asset reversibility compared to those in the eastern region. In these regions, corporate asset allocation exhibited higher liquidity and transaction clearing prices. However, asset reversibility had a more significant negative adjustment effect on corporate financial capital allocation in these regions. For instance, the mean asset reversibility in Inner Mongolia Autonomous Region was only 53.42%. Consequently, these companies were more prone to financial distress, and the moderating effect between asset reversibility and financial transaction assets in corporations was insignificant.

In technology-intensive sectors, technology is more liquidable and tradable compared to other production factors. When corporate asset reversibility is high, the intensity of deleveraging among listed companies can significantly inhibit the proportion of financial transaction assets. As a result of the decapacity policy, the asset reversibility adjustment effect is more significant for technology-intensive enterprises in the other three types of production factor-intensive industries. Therefore, the decapacity policy can significantly improve the degree of capital allocation in these industries.

### 5.3 Further discussion of the results

The deleveraging and decapacity policies, as the core of China’s supply-side structural reform during economic transition, significantly influence corporate capital allocation. The empirical results reveal several key findings. Firstly, deleveraging prompts enterprises to increase their financial asset allocation, and decapacity policies increase investment-related asset allocation. These two policy tools have distinct effects on corporate capital. Secondly, this study supports the mechanism of asset reversibility in the impact of deleveraging and decapacity on corporate capital allocation, indicating that asset reversibility plays a crucial role in the decision-making process of corporate asset allocation. Lastly, the heterogeneity analysis shows that asset reversibility can weaken the effect of the deleveraging policy more significantly on companies in eastern region while strengthening the effect of the decapacity policy more significantly on companies in central region. Moreover, asset reversibility plays a positive moderating role in the impact of decapacity on the capital allocation of technology-intensive companies.

This study can contribute to the field in two main aspects:

Firstly, in the context of promoting long-term sustainable economic development, we explored the factors that influence corporate asset allocation from the perspectives of leverage and capacity utilization. Studies based on endogenous growth theory have emphasized that a country’s long-term economic growth is not only determined by the high savings rate resulting from the constant marginal product of capital, but also by the crucial role of capital allocation (e.g., Bovenberg and Smulders [13]; Kraay and McKenzie [14]). As many developing countries worldwide are challenged by the middle-income trap (i.e., the input of capital factors fails to translate into equivalent output growth), identifying the driving force to overcome this growth barrier is essential for the long-term sustainable economic development. Thus, this study reveals the influencing factors that determine corporate capital allocation from leverage and capacity utilization. This can provide insights into achieving long-term sustainable economic development through structural optimization of asset allocation.

Secondly, based on real options theory, the concept of asset reversibility was introduced, which can measure corporate risk resilience. Existing literature has predominantly focused on the incentive effect of asset reversibility on corporate investment in an uncertain environment. It has been suggested that improved asset reversibility can enable enterprises to use the high liquidation value of investment projects to quickly overcome liquidity crises, thus maintaining relatively high investment levels in the face of heightened uncertainty regarding future expected returns (Campello and Giambona [58]; Gulen and Ion [19]; Kim and Kung [47]). However, previous studies have paid limited attention to the impact of asset reversibility on corporate capital allocation. This study can fill this gap and demonstrates that enhancing asset reversibility can weaken the inclination of companies to hold financial assets and strengthen their tendency to hold investment-related assets. Furthermore, these effects exhibit regional and industrial heterogeneity, thus enhancing the framework of asset reversibility and its relation to corporate asset allocation.

## 6. Conclusions and policy suggestions

Based on the policy background of the structural reform and sustainable development (referred to as "three cuts, one reduction and one addition"), the impacts of deleveraging and decapacity on corporate capital allocation were estimated. Three hypotheses were proposed and supported by the analysis results:

1. Implementing deleveraging and decapacity policies in China has a distinct impact on capital allocation. Specifically, for every 0.147 standard deviations increase in the degree of deleveraging, the average capital allocation of enterprises increases by 5.39%. In addition, the decapacity policy has a more significant effect on capital allocation, with every 0.209 standard deviations increase in production capacity enhancing capital allocation by 0.133% on average.
2. Considering the fundamental difference in the reversibility of corporate financial and capital asset investments, the deleveraging policy can weaken the inclination of companies to hold financial assets, while the decapacity policy can strengthen the capital investment of enterprises.
3. In term of China, differences between regional enterprises and geographical locations can also affect the effects of deleveraging and decapacity. Asset reversibility has a more pronounced weakening effect on enterprises in the eastern region, whereas the strengthening effect of decapacity is more significant on companies in the central region with capital assets. Furthermore, asset reversibility can positively mediate the impact of decapacity efforts on capital misallocation for technology-intensive companies.

Based on the above findings, this study has important policy implications for understanding the capital allocation adjustment during deleveraging and decapacity of listed companies:

1. Optimal utilization of policy tools: To achieve the optimal assignment of policy tools, deleveraging policies should be used to adjust holdings of corporate financial assets, while decapacity policies should be used to adjust holdings of corporate capital assets. This approach can provide strong policy support for enterprises, facilitating sustainable development and structural reform.
2. Inclusion of internal and external factors: Enterprise capital allocation is influenced by various internal and external factors. In the process of resolving overcapacity and alleviating high leverage ratios by implementing deleveraging and decapacity policies, it is important to pay attention to the transmission mechanism of asset reversibility. Throughout the enterprise life cycle, enterprises should gain experience in resource allocation based on the level of asset reversibility, adjust their capital structure and mitigate risks. This can enable them to avoid the challenges of insufficient asset liquidity and effectively resolve financial crises.
3. Importance of asset reversibility for technology-intensive enterprises: Technology-intensive enterprises should consider increasing their asset reversibility when faced with significant overcapacity reduction. When acquiring cutting-edge precision asset equipment, enterprises should consider the liquidation transaction price in the second-hand market. Strengthening asset reversibility can enable enterprises to expand their total investment and improve enterprise value. In addition, the geographical location can also affect the performance of deleveraging and decapacity policies. For example, in China, overburdened and overcapitalized enterprises in the central region can simultaneously increase their efforts in deleveraging and dcapacity. Proper utilization of financial leverage, activation of idle production capacity and investment in high-quality production capacity can enhance the ability of these enterprises to optimize their capital allocation.

## Supporting information

S1 File (DOCX)

S1 Data (XLSX)
