# Supplementary material for: Deleveraging and decapacity: A comparative analysis of corporate capital allocation based on asset reversibility
Source: PLoS One. 2023 Nov 15;18(11):e0291350. doi: 10.1371/journal.pone.0291350 (PMC10651045; doi:10.1371/journal.pone.0291350)
Supplement: S1 File — (DOCX) [file pone.0291350.s001.docx]

**Appendices**

**Appendix 1: Description of asset reversibility indicators by industry**

| Industry | Min | Max | Mean | Median |
| --- | --- | --- | --- | --- |
| Computing, communication, and other electronic equipment manufacturing | 0.467 | 109.5 | 61.85 | 70.47 |
| Automobiles | 0.007 | 112 | 64.2 | 69.31 |
| Equipment manufacturing | 0.029 | 893.2 | 63.15 | 68.64 |
| Raw Chemical Materials and Chemical Manufacturing | 0.382 | 124.7 | 60.1 | 66.73 |
| Paper and paper products | 22.81 | 65.01 | 58.95 | 61.84 |
| Electricity and heat production and supply | 7.245 | 101.8 | 55.91 | 61.23 |
| Metallurgical industry for non-ferrous metals | 6.965 | 68.89 | 54.52 | 61.21 |
| Metallurgical Mining and Processing | 31.94 | 62.76 | 53.85 | 58.41 |
| Civil Engineering and Construction | 0.161 | 81.98 | 53.68 | 57.76 |
| The Mining and Processing of Coal | 7.336 | 103.3 | 55.13 | 57.45 |
| Smelting and rolling of ferrous metals | 0.036 | 62.78 | 53.18 | 56.91 |
| Textured | 0.288 | 75.72 | 51.48 | 54.77 |
| Metals and nonferrous metals mining and processing | 5.903 | 95.63 | 52.08 | 52.9 |
| Manufacture of manufactured fibers | 12.16 | 79.41 | 50.72 | 52.39 |
| Oil refining, coking, and nuclear fuel processing | 0.161 | 78.9 | 44.63 | 49.27 |
| Petroleum and Gas Extraction | 15.14 | 53.09 | 37.51 | 38.19 |

Data: From China’s National Bureau of Statistics, authors own calculation

**Appendix 2: Statistics on asset reversibility indicators in each region of China**

| Province | Min | Max | Mean | Median |
| --- | --- | --- | --- | --- |
| Anhui | 0.467 | 73.43 | 60.65 | 64.45 |
| Beijing | 1.303 | 91.76 | 55.15 | 59.13 |
| Fujian | 5.245 | 73.43 | 56.28 | 59.53 |
| Gansu | 18.43 | 103.3 | 62.94 | 67.27 |
| Guangdong | 0.497 | 109.5 | 60.46 | 66.79 |
| Guangxi | 10.48 | 76.2 | 59.4 | 62.95 |
| Guizhou | 25.13 | 73.05 | 58.82 | 65.59 |
| Hainan | 8.915 | 112 | 54.6 | 53.09 |
| Hebei | 18.49 | 107.3 | 61.26 | 63.7 |
| Henan | 13.2 | 73.05 | 58 | 63.35 |
| Heilongjiang | 0.382 | 79.39 | 60.23 | 67.04 |
| Hubei | 4.186 | 893.2 | 60.42 | 63.39 |
| Hunan | 17.9 | 96.21 | 57.88 | 59.9 |
| Jilin | 12.16 | 74.52 | 61.4 | 66.22 |
| Jiangsu | 0.007 | 79.41 | 57.44 | 61.35 |
| Jiangxi | 20.97 | 73.43 | 57.02 | 62.9 |
| Liaoning | 0.036 | 73.43 | 57.07 | 59.66 |
| Inner Mongolia | 0.389 | 73.43 | 53.42 | 56.47 |
| Ningxia | 11.79 | 86.31 | 58.73 | 62.15 |
| Qinghai | 4.218 | 73.94 | 58.05 | 60.21 |
| Shandong | 0.13 | 75.89 | 59.54 | 63.52 |
| Shanxi | 0.161 | 73.05 | 53.53 | 56.98 |
| Shann xi | 19.37 | 73.43 | 56.35 | 61.89 |
| Shanghai | 0.029 | 103.8 | 55.81 | 59.93 |
| Sichuan | 0.161 | 101.8 | 57.22 | 60.84 |
| Tianjing | 20.66 | 73.19 | 60.22 | 69.97 |
| Xizang | 33.37 | 95.63 | 57.25 | 62.76 |
| Xinjiang | 1.231 | 124.7 | 54.07 | 57.62 |
| Yunnan | 25.09 | 78.9 | 55.37 | 63.4 |
| Zhejiang | 0.288 | 97.52 | 59.63 | 64.78 |
| Chongqing | 8.216 | 90.87 | 59.86 | 61.66 |

Data: From China’s National Bureau of Statistics, authors own calculation

**Appendix 3: The correlation coefficient matrix**

| Variable | *Fca* | *Cca* | *DLEV* | *RCEV* | *AR* | *Tq* | *Flrl* | *Size* | *Prfrm* | *Far* |
| --- | --- | --- | --- | --- | --- | --- | --- | --- | --- | --- |
| *Fca* | 1 |  |  |  |  |  |  |  |  |  |
| *Cca* | 0.0984 | 1 |  |  |  |  |  |  |  |  |
| *DLEV* | -0.061 | -0.162 | 1 |  |  |  |  |  |  |  |
| *RCEV* | -0.016 | -0.001 | -0.013 | 1 |  |  |  |  |  |  |
| *AR* | 0.0340 | 0.134 | 0.0184 | 0.061 | 1 |  |  |  |  |  |
| *Tq* | 0.135 | 0.147 | 0.0177 | 0.007 | 0.0897 | 1 |  |  |  |  |
| *Flr* | -0.115 | -0.643 | 0.0775 | 0.029 | -0.088 | -0.157 | 1 |  |  |  |
| *Size* | -0.267 | -0.509 | 0.0646 | 0.026 | -0.136 | -0.369 | 0.595 | 1 |  |  |
| *Prfrm* | 0.141 | 0.135 | 0.0121 | 0.016 | 0.0222 | 0.218 | -0.144 | -0.34 | 1 |  |
| *Far* | -0.282 | 0.207 | -0.096 | -0.025 | -0.012 | -0.116 | 0.071 | 0.182 | -0.034 | 1 |

**Appendix 4: Parallel trend test**

**
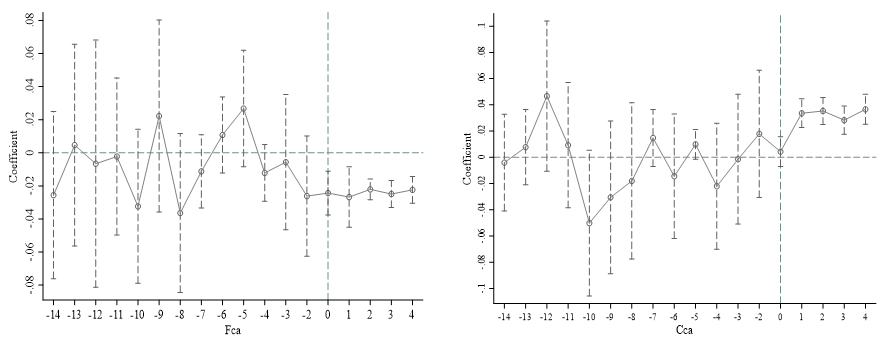
**

Notes: This figure shows the dynamic effects of the deleveraging and decapacity on corporate financial capital allocation and investment-related capital allocation. The x-axis is the time relative to the policy implementation time.

**Appendix 5: Placebo test**


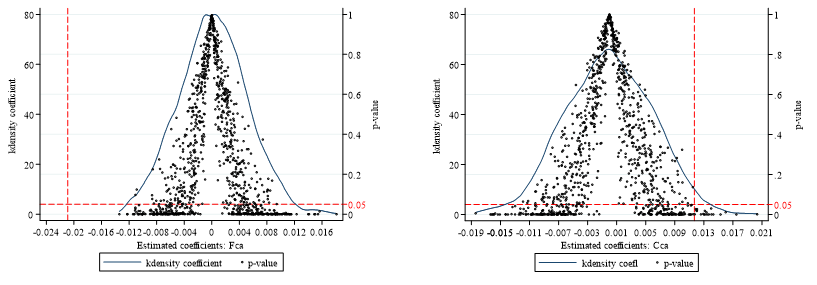


Notes: This figure shows the kernel density distributions and p-value scatter plots of the estimated coefficients for 1000 placebo tests with *Fca* and *Cca*, respectively. The mean of the estimated coefficients was about 0, significantly different from the real estimate indicated by the red vertical line. The majority of the estimated coefficients had p-values greater than 0.05 (indicated by the red horizontal line), confirming that the regression results of the DID approach was reasonable.
